# Disruptions in Brain Networks of Older Fallers Are Associated with Subsequent Cognitive Decline: A 12-Month Prospective Exploratory Study

**DOI:** 10.1371/journal.pone.0093673

**Published:** 2014-04-03

**Authors:** Chun Liang Hsu, Michelle W. Voss, Todd C. Handy, Jennifer C. Davis, Lindsay S. Nagamatsu, Alison Chan, Niousha Bolandzadeh, Teresa Liu-Ambrose

**Affiliations:** 1 Aging, Mobility, and Cognitive Neuroscience Lab, University of British Columbia, Vancouver, British Columbia, Canada; 2 Department of Physical Therapy, University of British Columbia, Vancouver, British Columbia, Canada; 3 Brain Research Center, University of British Columbia, Vancouver, British Columbia, Canada; 4 Center for Hip Health and Mobility, Vancouver, British Columbia, Canada; 5 Health, Brain, & Cognition Lab, University of Iowa, Iowa City, Iowa, United States of America; 6 Department of Psychology, University of Iowa, Iowa City, Iowa, United States of America; 7 Centre for Clinical Epidemiology and Evaluation, University of British Columbia & Vancouver Coastal Health Research Institute (VCHRI), Vancouver, British Columbia, Canada; 8 Department of Psychology, University of British Columbia, Vancouver, British Columbia, Canada; University Medical Center Groningen UMCG, Netherlands

## Abstract

Cognitive impairment and impaired mobility are major public health concerns. There is growing recognition that impaired mobility is an early biomarker of cognitive impairment and dementia. The neural basis for this association is currently unclear. We propose disrupted functional connectivity as a potential mechanism. In this 12-month prospective exploratory study, we compared functional connectivity of four brain networks– the default mode network (DMN), fronto-executive network (FEN), fronto-parietal network (FPN), and the primary motor sensory network (SMN) – between community-dwelling older adults with ≥ two falls in the last 12 months and their non-falling counterparts (≤ one fall in the last 12 months). Functional connectivity was examined both at rest and during a simple motor tapping task. Compared with non-fallers, fallers showed more connectivity between the DMN and FPN during right finger tapping (*p* = 0.04), and significantly less functional connectivity between the SMN and FPN during rest (*p*≤0.05). Less connectivity between the SMN and FPN during rest was significantly associated with greater decline in both cognitive function and mobility over the12-month period (r = −0.32 and 0.33 respectively; *p*≤0.04). Thus, a recent history of multiple falls among older adults without a diagnosis of dementia may indicate sub-clinical changes in brain function and increased risk for subsequent decline.

## Introduction

Cognitive impairment and impaired mobility among older adults are major public health concerns. Both are associated with increased risk for disability, institutionalization, and death [1]. Critically, there is growing recognition that clinical gait abnormalities and falls are early biomarkers of cognitive impairment and dementia [2]. For example, in the Health, Aging and Body Composition Study [3], slower gait speed at baseline was predictive of subsequent cognitive decline. Gait speed was also reported to slow at 0.023 meters/second/year approximately one decade before the diagnosis of mild cognitive impairment [4]. Conversely, baseline lower executive functions predicted subsequent decline in gait speed [5]. These results suggest cognitive decline and impaired mobility share common neurobiological mechanisms.

Neuroimaging studies highlight the role of white matter integrity in the association between cognitive function and mobility [6]–[9]. Both leukoaraiosis, or white matter lesions, and degeneration in white matter microstructure (i.e., myelodegeneration) are associated with cognitive decline and impaired mobility [10]–[15]. Functionally, white matter deterioration results in alterations in the coordination of brain networks that span multiple association cortices [16]–[18]. Functional connectivity analysis examines such disruptions; it aims to quantify the temporal coherence between spatially remote brain regions [19]. Regions with a positive correlation in blood-oxygen-level-dependent (BOLD) signal over time are said to have high functional connectivity, and regions uncorrelated or negatively correlated are thought to be in separate, or possibly competing, brain networks [20]. Current evidence suggests that a comprehensive examination of brain networks should include both task-free (“resting state”) and task-based conditions [21]. Mennes and colleagues [21] recently showed resting state connectivity patterns only correspond partially with on-task patterns. The authors found this is particularly true for sub-cortical regions, limbic regions, primary sensory cortex, and primary motor cortex.

A comprehensive examination of large-scale brain networks should also include both within-network and between-network functional connectivity [21]. To date, most studies have focused exclusively on within-network connectivity, particularly within the default mode network (DMN) [22]–[24]. Using neuroimaging data acquired from the “1000 Functional Connectomes Project” (http://www.nitrc.org/projects/fcon_1000/), Tomasi and colleagues [23] investigated the effect of aging on functional connectivity patterns within resting-state networks. They found that long range connections in the DMN and dorsal attention network were susceptible to aging-related deterioration. In a similar attempt to examine age-associated changes to intrinsic resting-state networks, Spreng and colleagues [25] demonstrated that older adults showed inability to suppress the DMN during cognitively active state, which was subsequently explained as reduced cognitive flexibility. However, recent evidence demonstrates that disruptions in both within-network and between-network connectivity can be observed during resting state [26], [27]. Specifically, using resting-state magnetic resonance imaging (MRI), Brier and colleagues [27] demonstrated that Alzheimer’s disease is associated with widespread loss of both within-network and between-network connectivity. Specifically, compared with non-demented older adults, individuals with very mild or mild Alzheimer’s disease exhibited less connectivity within the DMN and between five functionally defined networks.

**Figure 1:**
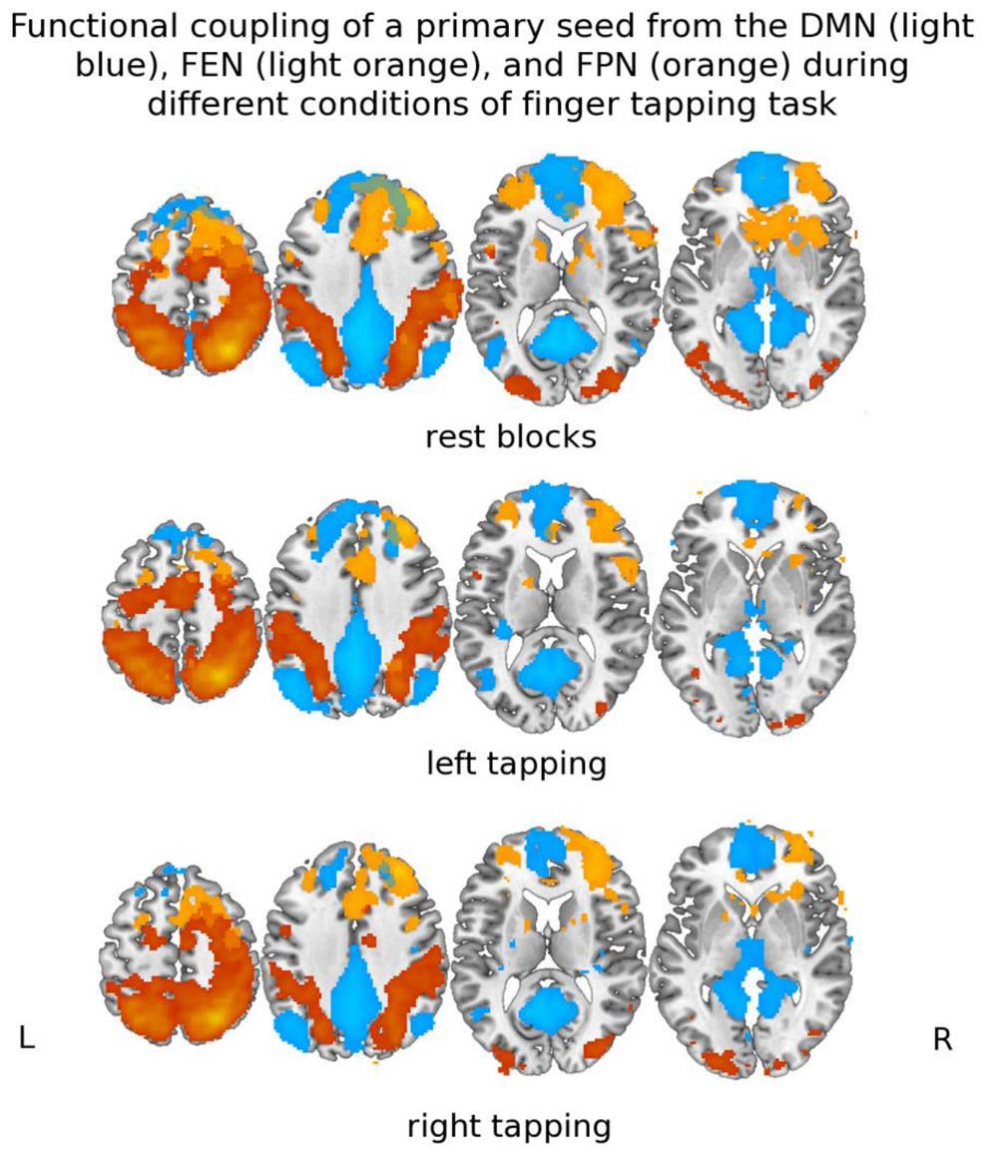
Group Functional Connectivity Map.

Aging and neurodegeneration are characterized by disruptions in the coordination of brain networks that support cognitive function and motor control [16], [28]–[31] [32]–[34]. These networks include the DMN, fronto-executive network (FEN), fronto-parietal network (FPN), and the primary motor sensory network (SMN) [16], [35]. The DMN is highly metabolically active when there is a lack of external stimulus (i.e., during rest) and deactivates during goal-oriented activity [36], [37]. Broadly, the DMN is involved in self-referential thoughts (i.e., accessing and processing of past events for the purpose of problem solving or future planning), memory consolidation, and autobiographical memory [16], [37]. The FEN is primarily involved in executive functions, error monitoring of top-down control, and maintaining an extended task-dependent cognitive state [38], [39]. The FPN is primarily involved in attentional control and contributes to cognitive abilities such as response anticipation and conflict processing [39]–[41]. The FPN and the SMN overlap in their anatomy (Figure 1) and both are involved in top-down control of motor planning and execution [32], [42], [43]. Critically, several studies revealed the FPN actively participate in the parcellation of long motor sequences into smaller motor segments [44], [45]– a process that improves motor performance accuracy and efficiency by reducing required overall cognitive load [46]. Older adults are less capable of performing motor sequence segmentation and researchers proposed this may be due to aging-related structural alteration in the FPN among the elderly [45]. Thus, examining the functional connectivity between these two networks may be of particular relevance to understanding the neural mechanisms underlying the association between cognitive function and mobility.

Given that impaired mobility precedes cognitive impairment and dementia [2], [4], and is significantly associated with white matter lesions [10], [11], [14], [47], we hypothesize that disrupted functional connectivity may be observed among older adults with impaired mobility without dementia and may be a neural mechanism underlying the association between reduced cognitive function and impaired mobility. Thus, the objectives of this exploratory study were: 1) to compare functional connectivity (within-network and between-network) of four brain networks, DMN, FEN, FPN, and SMN, using task-free and task-based conditions between community-dwelling older adults with a history of ≥2 falls in the last 12 months [48] (i.e., fallers) and their non-falling counterparts; and 2) to determine if differences in functional connectivity between fallers and non-fallers are associated with changes in cognitive function and mobility over a 12-month period. Specifically, we hypothesize that compared with non-fallers, fallers will demonstrate disrupted functional connectivity: 1) between the SMN and FPN during both task-free and task-based conditions; 2) between the SMN and FEN during task-based condition; and 3) within the DMN during task-free condition. Furthermore, we hypothesize that these differences in functional connectivity will be significantly associated with changes in cognitive function and mobility over 12 months. If our hypothesis is supported, a recent history of multiple falls among older adults without a diagnosis of dementia may be a biomarker of sub-clinical changes in brain function and increased risk for subsequent decline.

## Materials and Methods

### 2.1 Study Design and Participants

We conducted a 12-month prospective exploratory *s*tudy with 44 older adults. Participants were recruited from metropolitan Vancouver via newspaper advertisements. Individuals were eligible if they: 1) were aged 70 to 80 years; 2) scored ≥24/30 on the Mini-Mental State Examination (MMSE) [49]; 3) were right hand dominant as measured by the Edinburgh Handedness Inventory [50]; 4) were living independently in their own homes; 5) had visual acuity of at least 20/40, with or without corrective lenses; and 6) provided informed consent. We excluded those who: 1) had a neurodegenerative disease, stroke, dementia (of any type), or psychiatric condition; 2) had clinically significant peripheral neuropathy or severe musculoskeletal or joint disease; 3) were taking psychotropic medication; 4) had a history indicative of carotid sinus sensitivity; 5) were living in a nursing home, extended care facility, or assisted-care facility; or 6) did not meet MRI scanning requirements.

Based on their falls history in the 12 months prior to study entry, participants were classified as a faller or non-faller (see 2.1.1 and 2.1.2). Ethics approval was obtained from the Vancouver Coastal Research Health Institute and University of British Columbia’s Clinical Research Ethics Board. All participants provided written consent.

#### 2.1.1 Specific inclusion criterion for fallers

An individual must have experienced ≥2 minimal displacement non-syncopal falls in the previous 12 months, with one of the falls occurring in the last 6 months [51]. This was determined from two sources: 1) participant recall; and 2) participant’s immediate family member or friend recall. Falls were defined as “*unintentionally coming to rest on the ground, floor, or lower level*” [52].

#### 2.1.2 Specific inclusion criterion for non-fallers

An individual must not have experienced >1 displacement falls (with or without syncope) in the previous 12 months. This was determined based on two sources: 1) participant recall; and 2) participant’s immediate family member or friend recall. Individuals with one fall (non-injurious) in the previous 12 months resemble the physiological profile of non-fallers [48], [53]. Specifically, a prospective study found that while multiple falls (i.e., ≥2 falls) over 12 months were significantly associated with musculoskeletal and neurological deficits, single falls were not [48]. Importantly, older adults with a single fall were very similar to non-fallers in their physical and mental status.

### 2.2 Measurement

All measures, with the exception of neuroimaging, were assessed at baseline and 12 months. All assessors were trained and standardized protocols were used.

#### 2.2.1 Global cognition and current physical activity level

Global cognition was assessed using the MMSE [49] and the Montreal Cognitive Assessment (MoCA) [54]. The MoCA is a 30-point test that covers multiple cognitive domains. The MoCA has been found to have good internal consistency and test-retest reliability and was able to correctly identify 90% of a large sample of individuals with mild cognitive impairment from two different clinics with a cut-off scores of <26/30 [54]. Current level of physical activity (i.e., last 7 days) was determined by the Physical Activities Scale for the Elderly (PASE) self-report questionnaire [55].

#### 2.2.2 Comorbidity and depression

Comorbidities were assessed with the Functional Comorbidity Index (FCI) [56], a 21-item questionnaire that calculates the total number of comorbidities associated with physical functioning [56]. We used the 15-item Geriatric Depression Scale (GDS) [57], [58] to indicate the presence of depression; a score of ≥5 indicates depression [59].

#### 2.2.3 Falls-related self-efficacy

Self-efficacy is associated with cognitive function [60], [61], mobility [62]–[64], and brain volume [65]. In this study, we assessed falls-related self-efficacy on mobility-related tasks using the 16-item Activities-specific Balance Confidence (ABC) Scale [57]. Each item is rated from 0% (no confidence) to 100% (complete confidence) and a score out of 100 is calculated.

#### 2.2.4 Physiological falls risk

Physiological falls risk was assessed using the short form of the Physiological Profile Assessment (PPA). The PPA is a valid [58], [59] and reliable [60] measure of falls risk. Based on a participant’s performance in five physiological domains – postural sway, reaction time, strength, proprioception, and vision – the PPA computes a falls risk score (standardized score) that has a 75% predictive accuracy for falls among older people [53], [66]. A PPA Z-score of ≥0.60 indicates high physiological falls risk [67].

#### 2.2.5 Mobility and balance

Mobility and balance were assessed using the Short Physical Performance Battery (SPPB) [68] and the Timed-Up-and-Go Test (TUG) [69]. For the Short Physical Performance Battery, participants were assessed on performances of standing balance, walking, and sit-to-stand. Each component is rated out of four points, for a maximum of 12 points; a score <9/12 predicts subsequent disability [70]. For the TUG, participants rose from a standard chair, walked a distance of three meters, turned, walked back to the chair and sat down [69]. We recorded the time (s) to complete the TUG, based on the average of two separate trials.

#### 2.2.6 Executive functions

We used: 1) the Stroop Test [71] to assess selective attention and conflict resolution; 2) the Trail Making Tests (Part A & B) to assess set shifting [72]; and 3) the verbal digits forward and backward tests to index working memory [73]. For the Stroop Test [71], participants first read out words printed in black ink (e.g., BLUE). Second, they named the display colour of coloured-X’s. Finally, they were shown a page with colour-words printed in incongruent coloured inks (e.g., the word “BLUE” printed in red ink). Participants were asked to name the ink colour in which the words were printed (while ignoring the word itself). We recorded the time participants took to read the items in each condition and calculated the time difference between the third condition (Stroop 3) and the second condition (Stroop 2). Smaller time differences indicate better selective attention and conflict resolution performance.

For the Trail Making Tests (Part A & B) [74], participants were required to draw lines connecting encircled numbers sequentially (Part A) or alternating between numbers and letters (Part B). The difference in time to complete Part B and Part A was calculated, with smaller difference scores indicating better set shifting performance.

For the Verbal Digits Forward and Backward Tests [75], participants repeated progressively longer random number sequences in the same order as presented (forward) and the reversed order (backward). Successful performance on the verbal digits span backward test represents a measure of central executive function due to the additional requirement of manipulation of information within temporary storage [76]. Thus, we subtracted the verbal digits backward test score from the verbal digits forward test score to provide an index of working memory with smaller difference scores indicating better working memory.

### 2.3 Functional MRI (fMRI)

All fMRI was performed at the UBC MRI Research Center located at the UBC Hospital on a 3.0 Tesla Intera Achieva MRI Scanner (Phillips Medical Systems Canada, Markham, Ontario) using an 8-channel SENSE neurovascular coil. The fMRI consisted of 166 dynamic images of 36 slices (3 mm thick) with the following parameters: repetition time (TR) of 2000 milliseconds (ms), echo time (TE) of 30 ms, flip angle (FA) of 90 degrees, field of view (FoV) of 240 mm, acquisition matrix 80×80. The high resolution T1 images were acquired using the following parameters: 170 slices (1 mm thick), TR of 7.7 ms, TE of 3.6 ms, FA of 8 degrees, FoV of 256 mm, acquisition matrix of 256×200.

#### 2.3.1 Motor task

During the fMRI scan, participants performed a simple finger tapping motor test that allows the examination of functional connectivity of networks both during rest and on task. This type of motor task is sensitive in differentiating older adults with Alzheimer’s disease from those with other types of dementia [77].

Our motor task consisted of three conditions: left finger tapping, right finger tapping, and rest. The participants performed finger tapping with the respective hands as indicated, starting with index finger and progressing outward to the little (pinky) finger. This finger tapping motion was continuously performed until the next condition was displayed. For the rest condition, the participants were asked to rest with their eyes open.

The specific order of the motor task blocks, which was not disclosed to the study participants, was counter-balanced over three runs as followed:

Run 1: Rest, Left, Rest, Right, Rest, Left, Rest, Right, Rest

Run 2: Rest, Right, Rest, Left, Rest, Right, Rest, Left, Rest

Run 3: Rest, Left, Rest, Left, Rest, Right, Rest, Right, Rest

Each run contained nine short blocks of 34 seconds, and the duration of each run was 330.897 seconds.

### 2.4 Data Analysis

#### 2.4.1 Functional MRI data preprocessing

Image preprocessing was carried out using tools from FSL (FMRIB’s Software Library) [78], MATLAB (Matrix Laboratory), and toolboxes from SPM (Statistical Parametric Mapping). Excess unwanted structures (i.e., bones, skull, etc.) in high resolution T1 images were removed via Brain Extraction Tool (BET); rigid body motion correction was completed using MCFLIRT (absolute and relative mean displacement were subsequently extracted and included in the statistical analysis as covariates); spatial smoothing was carried out using Gaussian kernel of Full-Width-Half-Maximum (FWHM) 6.0 mm; temporal filtering was applied with high pass frequency cut-off of 120 seconds. In addition, a low pass temporal filtering was also included to ensure the fMRI signal fluctuated between 0.008<f<0.080 Hz, the ideal bandwidth to examine functional connectivity. Furthermore, the application of a low pass filter eliminated high frequency signals that could be confounds. Participants’ low-resolution functional data were registered to personal high resolution T1 anatomical images, which were subsequently registered to standardized 152 T1 Montreal Neurological Institute (MNI) space.

Noise generated from both physiological and non-physiological sources were removed through regression of the cerebral-spinal fluid (CSF) signal, white matter signal, and global brain signal. In addition, excess movement of the study participant was corrected by regression of motion parameters.

#### 2.4.2 Functional connectivity analysis

Previous studies guided our choice of seeds in the whole brain analysis of the DMN, FEN, FPN, and SMN [16], [32], [35]. The DMN included the posterior cingulate cortex (PCC), ventral and superior frontal medial cortices (FMC), middle temporal gyrus (MTG), parahippocampal gyrus (PHG), middle frontal gyrus (MFG), and lateral occipital cortex (LOC) [20], [37]. The FEN included the anterior lateral prefrontal cortex (RALPFC), insular sulcus (INS), prefrontal cortex (PFC), inferior frontal gyrus (IFG), and anterior cingulate gyrus (CING) [38]. The FPN included the inferior parietal sulcus (IPS), ventral visual cortex (VV), supramarginal gyrus (SMG), superior lateral occipital cortex (SLOC), frontal eye field (FEF), as well as overlapping areas in the temporal-parietal junction [38]. The SMN included the primary motor cortex (PCG), cerebellum (CB), premotor area (PM), and supplementary motor area (SMA) [32]. We examined the left and right SMN individually because the use of dominant or non-dominant hand evokes different neural activity between the hemispheres [79]. Thus, examining each hemispheric SMN separately provides a better understanding of their temporal coherence with other networks during finger tapping. The respective MNI space coordinates for each region of interest (ROI) are provided in Table 1.

**Table 1:** Neural Networks and Regions of Interests Included in the Analysis.

Neural Networks†	Region of Interest	MNI Coordinates (mm)		
		X	Y	Z
DMN	PCC	8	−56	30
	FMC	−2	54	−12
	RMTG	58	−10	−18
	LMTG	−52	−14	−20
	RPHG	24	−26	−20
	LPHG	−26	−24	−20
	LMFG	−30	20	50
	RLOC	54	−62	32
	LLOC	−44	−72	30
FEN	RALPFC	32	40	28
	RINS	38	4	−2
	LINS	−38	8	−4
	RPFC	32	42	36
	LPFC	−36	34	28
	RIFG	34	48	−6
	LIFG	−38	48	8
	CING	4	28	26
FPN	RIPS	25	−62	53
	RVV	36	−62	0
	LVV	−44	−60	−6
	RSMG	32	−38	38
	RSLOC	26	−64	54
	LSLOC	−26	−60	52
	RFEF	28	−4	58
	LFEF	−26	−8	54
SMN	LPCG	−39	−21	55
	RPCG	34	−25	53
	LCB	−24	−66	−19
	RCB	25	−71	−23
	LPM	−16	0	57
	RPM	20	−17	61
	SMA	−5	−1	52

† PCC = posterior cingulate cortex; FMC = frontal medial cortex; RMTG/LMTG = right/left middle temporal gyrus; RPHG/LPHG = right/left parahippocampal gyrus; LMFG =  left middle frontal gyrus; RLOC/LLOC = right/left parietal cortex; RALPFC = right anterior lateral prefrontal cortex; RINS/LINS =  right/left insular sulcus; RPFC/LPFC =  right/left prefrontal cortex; RIFG/LIFG =  right/left inferior frontal gyrus; CING =  cingulate; RIPS = right inferior parietal sulcus; RVV/LVV =  right/left ventral visual; RSMG = right supramarginal gyrus; RSLOC/LSLOC =  right/left occipital cortex; RFEF/LFEF =  right/left frontal eye field; LPCG = left precentral gyrus; RPCG = right precentral gyrus; LCB = left cerebellum; RCB = right cerebellum; LPM = left premotor; RPM = right premotor; SMA = supplementary motor area.

From each ROI, preprocessed time-series data were extracted with 14 mm spherical regions of interest drawn around their respective MNI coordinates in standard space. The different conditions (i.e., left, right, and rest) within each block of the motor task were extracted and compiled together [80]. To concatenate the time-series data, the stimulus onset time for each task condition was acquired from the task program. Each volume of the data was then sorted according to their respective condition. Once the data were properly categorized, the task-specific volumes (e.g. all the “left” volumes) were merged using a bash script provided in the FSL program. The first three volumes of any condition were discarded to account for delay of the hemodynamic response. To ensure our results were not affected by small number of time points in the fMRI data, we performed an addition analysis in which all task conditions were combined (please see Text S1 and Table S1 in Supplementary S1).

Region of interest time-series data were subsequently cross-correlated with every voxel within the brain to establish functional connectivity maps of their associated neural networks. Individual-level within-subject results were generated via ordinary least squares (OLS) in FSL by congregating the voxel-wise functional connectivity maps from each condition. Similarly, for group results, a mixed-level OLS analysis was conducted. The statistical map thresholding was set at Z = 2.33, with cluster correction of *p*<0.05. Pearson’s correlation coefficients were subsequently calculated between the ROI listed in Table 1.

#### 2.4.3 Statistical analysis

To normalize our data, the Pearson’s correlation coefficients (between the time-series of the seeded region and other voxels in the brain) were converted into Fisher’s z correlation coefficients via Fisher’s r-to-z transformation [81] in MATLAB. Fisher’s transformation generates normally distributed sample distribution and ensures the variance of the correlation coefficient remain constant for all values in the sample population correlation [81].

To reduce Type I error and potentially produce a more robust signal, we reduced the number of comparisons by averaging all the ROI-pairs within each of the four networks as well as all ROI-pairs between the networks. For example, the DMN contains the following 6 ROIs: PCC, FMC, RMTG, RPHG, LMFG, and RLOC. Six ROIs result in 15 ROI-pairs. To calculate the mean network correlation for the DMN, we totalled the 15 Fisher’s z correlation coefficients and then divided the total value by 15.

Data were analyzed using the IBM SPSS Statistic 19 for Windows (SPSS Inc., Chicago, IL). Descriptive data are reported for variables of interest. Comparisons of group characteristics at baseline were undertaken using a Chi Square test for differences in proportions and ANOVAs for differences in means. Analysis of covariance (ANCOVA) was performed to statistically test for significant between-group differences in mean network functional connectivity. In the model, baseline FCI, baseline mean ABC Scale score [62]–[64], and mean relative head motion (extracted from McFLIRT) [82], [83] were included as covariates. The overall alpha value was set at *p*≤0.05. Overall, 18 within-network and 27 between-network group comparisons in functional connectivity were performed.

Finally, Pearson correlations were computed to determine whether significant differences in mean network functional connectivity observed at baseline between fallers and non-fallers were significantly associated with changes in cognitive function and mobility over a 12-month period. Change for all measures of cognitive function and mobility was calculated as: 12-month value minus baseline value. For example, change in Stroop Test performance was calculated as: (12-month Stroop 3 completion time –12-month Stroop 2 completion time) – (baseline Stroop 3 completion time – baseline Stroop 2 completion time). Hence, for the Stroop Test, negative change values reflect improvement. Conversely, positive change values for SPPB reflect improvement. Overall, 36 correlation comparisons were conducted.

## Results

### 3.1 Participant

Of the 44 participants, 23 were classified as fallers and 21 were classified as non-fallers. Among the 21 non-fallers, six participants had 1 falls in the prior 12 months. Number of falls 12-month prior to the study ranged from 2–20 in the fallers group; 0–1 in the non-fallers group. Other than the number of falls reported in the previous 12 months, fallers and non-fallers were not significantly different at baseline, including falls risk as measured by the PPA (Table 2). Based on the mean MoCA scores, our participants had mild cognitive impairment. Notably, the proportion of individuals with MoCA scores <26/30 was not significantly different between the two groups. No differences between the two groups were detected 12 months later (Table 3).

**Table 2:** Baseline Description of Participants.

Variable†	Group (n = 44)		Fallers (n = 21)		Non-fallers (n = 23)		*p*-value
	Mean	SD	Mean	SD	Mean	SD	
Gender (m/f)	9/35		3/18		6/17		0.55
Age (years)	74.34	3.21	74.21	3.33	74.44	3.18	0.58
Falls in the Prior 12 Months 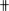	1.66	3.07	3.19	3.92	0.26	0.45	**<0.01**
MoCA (30 pts)	24.77	3.12	24.50	2.89	25.00	3.35	0.39
MMSE (30 pts)	28.20	1.49	28.20	1.64	28.21	1.38	0.75
Stroop Test (seconds)	55.06	25.38	55.60	27.31	54.55	24.09	0.89
Trail Making (seconds)*	62.91	122.18	83.63	171.83	44.00	39.41	0.90
Digits Forward/Backward	3.68	2.15	2.95	2.04	4.35	2.08	0.08
Functional Comorbidity Index	2.93	1.86	3.10	1.80	2.79	1.93	0.48
Geriatric Depression Scale	0.48	0.90	0.57	1.03	0.39	0.78	0.67
Activities-Specific Balance Confidence scale (max 100%)	83.61	16.76	79.30	21.75	87.20	10.25	0.16
Timed Up and Go (seconds)	7.92	2.94	8.66	4.13	7.31	1.09	0.35
Physiological Profile Assessment (z-score)	0.35	0.86	0.60	0.87	0.15	0.81	0.10
Short Physical Performance Battery (max 12 pts)	10.14	1.83	9.71	1.79	10.52	1.81	0.48
4-Meter Gait Speed (m/s)	1.27	0.24	1.22	0.04	1.31	0.04	0.14
Mean Absolute Displacement (mm)	-	-	0.50	0.22	0.61	0.36	0.22
Mean Relative Displacement (mm)	-	-	0.20	0.09	0.19	0.11	0.63
Number of Individual with MOCA <26/30 (i.e. MCI)	21/44		11		10		0.76

† m, male; f, female; MMSE, Mini Mental State Examination; MoCA, Montreal Cognitive Assessment; Stroop Test = Stroop 3– Stroop 2, units in seconds; Trail Making = Trail Making B – Trail Making A, units in seconds; Digits Forward/Backward = Digits Backward – Digits Forward; m/s = meters/second; MCI, Mild Cognitive Impairment.

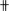
Number of falls 12-month prior to study ranged from 2–20 in the Fallers group; 0–1 in the Non-Fallers group.

*Statistical analysis included one outlier with 816.94 seconds on the Trail Making; after removal of this individual same relationships persist across the variables (*p*<0.01 for Falls in previous 12 months; *p*>0.05 for other variables).

**Table 3:** Twelve-Month Follow-Up Description of Participants.

Variable†	Group (n = 44)		Fallers (n = 21)		Non-fallers(n = 23)		*p*-value
	Mean	SD	Mean	SD	Mean	SD	
Gender (m/f)	9/35		3/18		6/17		0.55
Age (years)	74.34	3.21	74.21	3.33	74.44	3.18	0.58
Number of Falls During Study 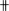	1.16	1.54	1.43	1.96	0.91	1.19	**<0.01**
MoCA (30 pts)	23.65	3.89	22.90	3.35	24.80	3.32	0.08
MMSE (30 pts)	28.00	1.90	28.10	1.86	28.05	1.73	0.93
Stroop Test (seconds)	49.78	24.74	49.37	19.00	50.13	29.26	0.92
Trail Making (seconds)	38.92	24.04	40.59	24.68	37.47	25.07	0.69
Digits Forward/Backward	3.56	2.22	3.65	2.35	3.50	2.06	0.83
Timed Up and Go (seconds)	7.24	1.74	7.73	1.30	7.16	1.34	0.18
Physiological Profile Assessment (z-score)	0.11	0.99	0.25	1.04	−0.15	0.93	0.21
Short Physical Performance Battery (max 12 pts)	10.81	1.84	10.80	1.40	11.40	1.10	0.14
4-Meter Gait Speed (m/s)	1.17	0.19	1.15	0.20	1.21	0.16	0.26
Number of Individual with MOCA <26/30 (i.e. MCI)	26/44		14		12		0.24

† m, male; f, female; MMSE, Mini Mental State Examination; MoCA, Montreal Cognitive Assessment; Stroop Test = Stroop 3– Stroop 2, units in seconds; Trail Making = Trail Making B – Trail Making A, units in seconds; Digits Forward/Backward = Digits Backward – Digits Forward; m/s = meters/second; MCI, Mild Cognitive Impairment.

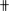
Number of falls during study ranged from 0–7 in the Fallers group; 0–4 in the Non-Fallers group.

### 3.2 Functional Connectivity of Fallers versus Non-fallers

#### 3.2.1 Mean within-network connectivity

There were no significant differences in within-network connectivity between fallers and non-fallers (Table 4). Overall, compared with non-fallers, fallers exhibit a non-significant trend for less functional connectivity within each network across all conditions (*p*≥0.12).

**Table 4:** Within-Network Functional Connectivity Results.

Network†	Task Condition	Fallers		Non-fallers		*p*-value*
		Mean	SD	Mean	SD	
DMN	Rest	0.22	0.14	0.24	0.17	0.61
	Right	0.24	0.17	0.30	0.16	0.32
	Left	0.26	0.16	0.25	0.20	0.83
FEN	Rest	0.20	0.10	0.19	0.11	0.58
	Right	0.17	0.07	0.15	0.13	0.49
	Left	0.15	0.11	0.16	0.16	0.95
FPN	Rest	0.28	0.14	0.32	0.17	0.31
	Right	0.28	0.19	0.35	0.22	0.15
	Left	0.31	0.16	0.33	0.22	0.48
Left Hemispheric SMN	Rest	0.23	0.17	0.28	0.19	0.39
	Right	0.22	0.21	0.29	0.14	0.16
	Left	0.22	0.21	0.23	0.19	0.42
Right Hemispheric SMN	Rest	0.20	0.15	0.23	0.17	0.44
	Right	0.21	0.22	0.21	0.17	0.88
	Left	0.20	0.18	0.27	0.19	0.12

† DMN = default mode network; FEN = fronto-executive network; FPN = fronto-parietal network; SMN = motor network.

*Controlled for FCI, mean ABC scale score, and relative head motion.

#### 3.2.2 Mean between-network connectivity

**Figure 2:**
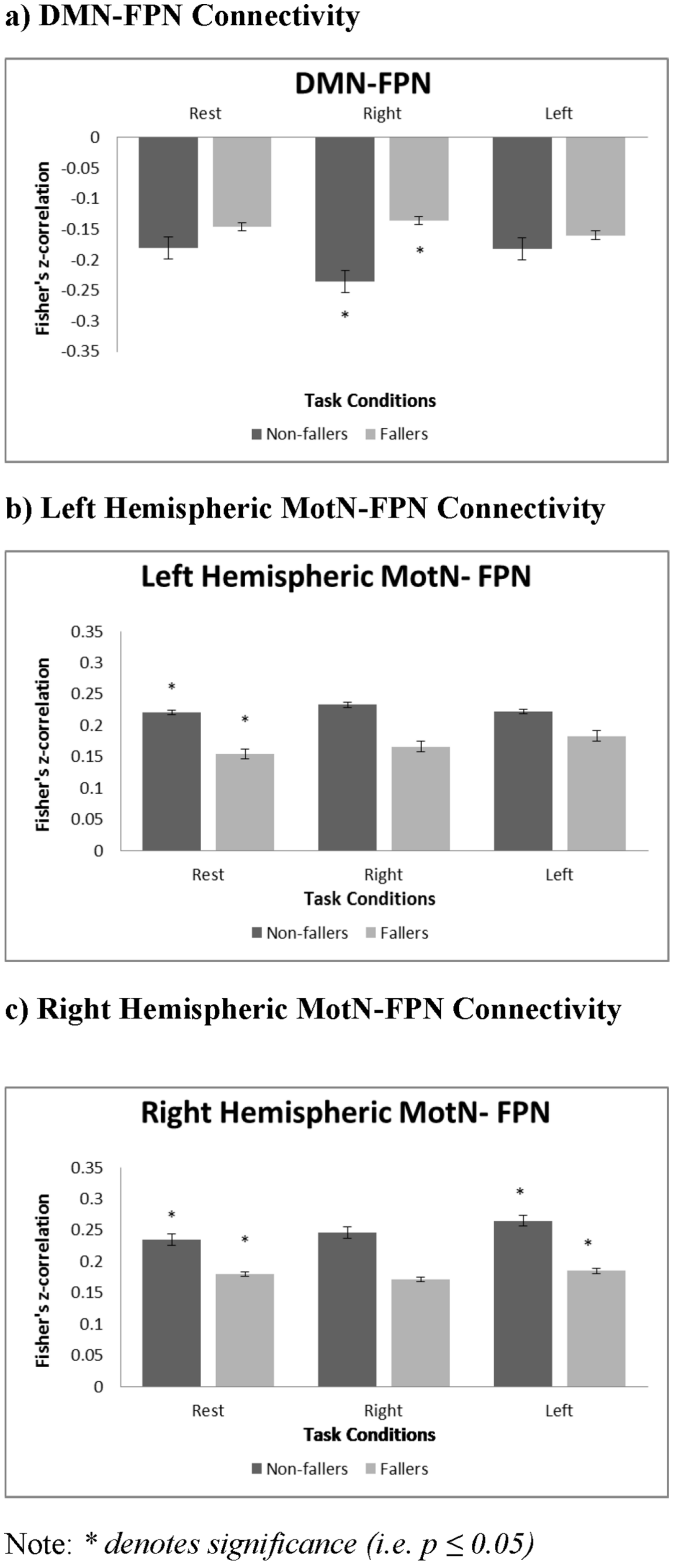
Graphical Representations of Between-Network Connectivity Significantly Different Between Fallers and Non-Fallers.

Figure 2 reports the between-network connectivity correlation coefficients. Compared with non-fallers, fallers showed greater connectivity between the DMN and FPN during right hand finger tapping (*p* = 0.04) (Figure 2a; Table 5). Furthermore, Compared with non-fallers, fallers demonstrated significantly less connectivity between the left hemispheric SMN and FPN (*p* = 0.02) during rest (Figure 2b; Table 5), and between the right hemispheric SMN and FPN during rest (*p* = 0.05) and left hand finger tapping (*p* = 0.03) (Figure 2c; Table 5).

**Table 5:** Between-Network Functional Connectivity Results.

Network Pairs†	Task Condition	Fallers		Non-fallers		*p*-value *
		Mean	SD	Mean	SD	
DMN-FEN	Rest	−0.02	0.08	−0.02	0.08	0.88
	Right	−0.04	0.11	−0.03	0.08	0.36
	Left	−0.04	0.11	−0.05	0.09	0.70
DMN-FPN	Rest	−0.15	0.11	−0.18	0.14	0.38
	**Right**	**−0.14**	**0.14**	**−0.24**	**0.18**	**0.04** †
	Left	−0.16	0.13	−0.18	0.14	0.64
FEN-FPN	Rest	−0.02	0.08	−0.01	0.10	0.88
	Right	<−0.01	0.11	<−0.01	0.14	0.71
	Left	−0.01	0.10	0.01	0.10	0.60
Left Hemispheric SMN-DMN	Rest	−0.14	0.10	−0.17	0.14	0.41
	Right	−0.14	0.10	−0.18	0.13	0.19
	Left	−0.18	0.11	−0.16	0.13	0.62
Left Hemispheric SMN-FEN	Rest	0.02	0.09	0.02	0.08	0.96
	Right	0.04	0.10	0.01	0.15	0.50
	Left	<0.01	0.11	0.05	0.09	0.14
Left Hemispheric SMN-FPN	**Rest**	**0.15**	**0.09**	**0.22**	**0.12**	**0.02** †
	Right	0.17	0.13	0.23	0.14	0.09
	Left	0.18	0.10	0.22	0.17	0.24
Right Hemispheric SMN-DMN	Rest	−0.15	0.12	−0.17	0.13	0.78
	Right	−0.14	0.13	−0.16	0.13	0.49
	Left	−0.16	0.11	−0.18	0.13	0.55
Right Hemispheric SMN-FEN	Rest	0.02	0.09	<−0.01	0.08	0.28
	Right	0.02	0.11	−0.03	0.15	0.18
	Left	0.01	0.08	<−0.01	0.13	0.84
Right Hemispheric SMN-FPN	**Rest**	**0.18**	**0.11**	**0.23**	**0.10**	**0.05** †
	Right	0.17	0.15	0.25	0.12	0.07
	**Left**	**0.18**	**0.12**	**0.26**	**0.16**	**0.02** †
Right-Left Hemispheric Mot	Rest	0.16	0.18	0.16	0.25	0.82
	Right	0.15	0.19	0.15	0.19	0.71
	Left	0.12	0.21	0.07	0.20	0.36

† DMN = default mode network; FEN = fronto-executive network; FPN = fronto-parietal network; SMN = motor network.

*p≤0.05; controlled for FCI, mean ABC scale score, and relative head motion.

#### 3.2.3 Correlation results

**Figure 3:**
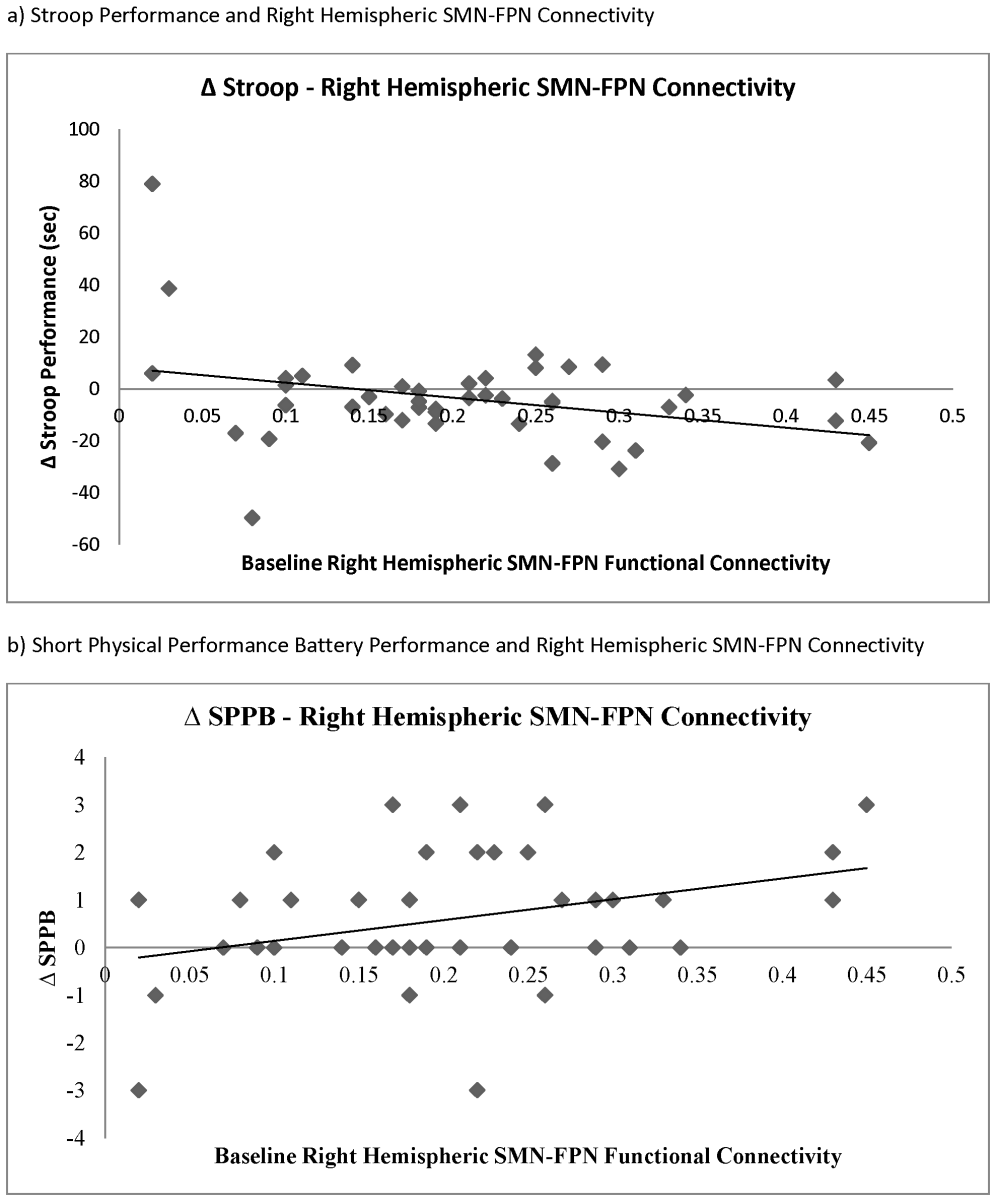
Correlations of Behavioural Performances and Functional Connectivity.

We found connectivity between the right hemispheric SMN and FPN during rest was significantly associated with change in Stroop Test performance (Pearson’s r = −0.32, *p* = 0.04*;* Spearman’s r =  −0.21, *p* = 0.18) Table 6; Figure 3a) and SPPB performance (Pearson’s r = 0.33, *p* = 0.03; Spearman’s r =  −0.27, *p* = 0.09; Table 6; Figure 3b). Specifically, less connectivity between the right hemispheric SMN and FPN during rest, as demonstrated by fallers at baseline, was associated with greater reductions in Stroop Test and SPPB performance over the 12-month study period. As indicated in Figure 3a, the association between the right hemispheric SMN and FPN during rest and change in Stroop Test performance may be influenced by an extreme (positive) score. Hence, we also calculated the Spearman correlation, which was r = −0.21 (p = 0.18).

**Table 6:** Correlations Between Change in Performance Measures and Baseline Functional Connectivity.

Variable†	Mean Change		Right Hemispheric SMN-FPN at Rest		Right Hemispheric SMN-FPN Left Finger Tapping		Left Hemispheric SMN-FPN at Rest		DMN-FPN Right Finger Tapping	
	Mean	SD	Pearson	Spearman	Pearson	Spearman	Pearson	Spearman	Pearson	Spearman
ΔMoCA (max of 30)	−1.12	2.53	0.30	0.26	0.09	0.00	0.13	0.02	−0.24	−0.22
ΔStroop Test (seconds)	−3.55	19.08	**−0.32** *	−0.21	−0.15	−0.12	−0.14	−0.11	−0.09	−0.11
ΔTrail Making Test (seconds)	−4.29	30.46	0.21	−0.11	0.20	−0.23	0.10	−0.24	−0.09	0.11
ΔDigits Forward and Backward Test	−0.23	3.00	0.02	0.07	0.17	0.23	−0.17	−0.01	−0.08	−0.03
ΔTimed Up and Go (seconds)	−0.61	3.04	−0.02	−0.04	0.26	−0.00	0.03	−0.08	−0.34	0.08
ΔPhysiological Profile Assessment	−0.21	0.91	0.23	0.19	0.15	0.12	0.21	0.12	−0.19	0.04
ΔShort Physical Performance Battery	0.60	1.38	**0.33** *	0.27	0.26	0.19	0.11	0.11	−0.03	0.06
Δ4-Meter Gait Speed (m/s)	−0.11	0.20	0.09	−0.03	0.10	0.01	0.23	−0.08	0.20	0.15

† MMSE, Mini Mental State Examination; MoCA, Montreal Cognitive Assessment; Stroop Test = Stroop 3– Stroop 2, units in seconds; Trail Making = Trail Making B – Trail Making A, units in seconds; Digits Forward/Backward = Digits Backward – Digits Forward; m/s = meters/second; MCI, Mild Cognitive Impairment.

Change calculated as: 12-month value minus baseline value.

Negative value represents improvement for: ΔStroop Test, ΔTrail Making Test, ΔTimed Up and Go, ΔDigits Forward and Backward Test, ΔPhysiological Profile Assessment.

Positive value represents improvement for: ΔMoCA, ΔMMSE, ΔShort Physical Performance Battery, Δ4-Meter Gait Speed.

*p≤0.04.

## Discussion

Findings from our exploratory study suggest that community-dwelling older fallers without dementia may have disrupted functional connectivity between large neural networks. Specifically, compared with non-fallers, fallers demonstrated greater connectivity between the DMN and FPN during right hand finger tapping and less connectivity between the SMN and FPN during rest and left hand finger tapping. Importantly, less connectivity between the right hemispheric SMN and FPN during rest was significantly associated with greater decline in mobility over the 12-month period. There was also suggestion that less connectivity between the right hemispheric SMN and FPN during rest was associated with greater decline in cognitive function as measured by the Stroop Test. Thus, a recent history of multiple falls among older adults without a diagnosis of dementia may indicate sub-clinical changes in brain function and increased risk for subsequent decline.

The finding of increased connectivity between the DMN and FPN among older fallers during right hand finger tapping condition provides novel insight into the neural risk factors for falls. It is widely recognized that the DMN deactivates during goal-oriented activity [36], [37]. In accord with this concept, evidence in the literature found an association between reduced cognitive performance and aging-related increase in functional connectivity between the DMN and FPN [84]. Thus, our results suggest that fallers may have reduced ability to disengage from internally-generated thoughts when performing a task. In particular, our participants were right hand dominant and thus, right finger tapping was less cognitively demanding than left finger tapping [85]. According to the capacity model of attention, during a less cognitively-demanding task, more resources are available to distribute to task-irrelevant stimuli or thoughts [86]. Hence, we speculate that the reduced ability to attend externally by disengaging from thoughts focused internally, or increased propensity for mind-wandering, may contribute to falls risk among older adults. Certainly there is emerging evidence to suggest that mind-wandering negatively impacts motor control [87], [88].

Majority of the differences we found in functional connectivity were between the SMN and FPN. This supports our original hypothesis that these two functionally- and anatomically-overlapping networks (Figure 1) are of specific interest in understanding the neural basis for the association between cognitive function and mobility. Our observation that connectivity between the SMN and FPN during rest was less among fallers than non-fallers concurs and extends previous findings. Inman and colleagues [89] also found less connectivity between the SMN and FPN during rest in stroke survivors – a population that is also at significant risk for falls and dementia [90]–[92]. Less connectivity between these two networks during rest may suggest reduced motor preparatory inputs, in anticipation of motor performance, from FPN to the SMN. This, in turn, may increase falls risk. Our fMRI design may have been particularly sensitive to evoking anticipation because the rest periods were interspersed among the finger tapping blocks. Using event related potentials, Berchicci and colleagues [93] recently demonstrated that older adults require greater motor preparation (as indexed by earlier onset latency onset and greater prefrontal cortex activation) than young adults to obtain the same level of motor performance.

Compared with non-fallers, fallers also demonstrated less connectivity between the right hemispheric SMN and FPN during left finger tapping. Evidence suggests that compared with dominant hand use, non-dominant hand use requires greater neural resources (bilateral recruitment of the brain) to maintain stable motion [79], [85]. Given our participants were right hand dominant, one would expect greater connectivity between the right hemispheric SMN and FPN during left finger tapping than right finger tapping. Importantly, we did observe the mean network connectivity between right hemispheric SMN and FPN during left finger tapping (i.e., 0.19) was greater than the mean network connectivity left hemispheric SMN and FPN right finger tapping (i.e., 0.17). However, these connectivity values were not significantly different (p = 0.78). Thus, this finding suggests that reduced temporal coherence between the SMN and FPN during novel or challenging motor tasks may increase falls risk among older adults.

Furthermore, we found that less functional connectivity between the right hemispheric SMN and FPN during rest was significantly associated with greater decline in both cognitive function and mobility over the 12-month period. This finding further supports the hypothesis that both cognitive decline and impaired mobility may share common neurobiological mechanisms.

We did not observe any significant group differences in the functional connectivity between the SMN and FEN. This null finding could be because the motor task does not specifically engage executive processes, such as working memory or maintenance of a complex task set. We also did not observe any significant group differences within the DMN. Changes within the DMN have been observed in healthy aging [22], [94], [95], Alzheimer’s disease [34], [95], [96], individuals with amnestic mild cognitive impairment [97], and in APOE-*ε*4 carriers [98], [99]. Hence, the lack of significant differences between fallers and non-fallers in age, MoCA score, MMSE score, and executive functions, may explain our null finding within the DMN. Our findings may also suggest that between-network functional connectivity disruptions may precede within-network connectivity disruptions in older adults without a diagnosis of dementia. However, future studies are needed to confirm the temporal order of functional connectivity disruptions with both healthy aging and neurodegeneration.

It is noteworthy that despite observing significant differences in functional connectivity between fallers and non-fallers, we found no differences in measures of falls risk, mobility and balance, and cognitive function. Importantly, based on the performance on the MoCA, the number of mild cognitively impaired individuals was equally distributed between fallers and non-fallers (Table 2). The only clinical characteristic that significantly distinguished the two groups was the number of falls in the 12 months prior to enrollment in the study. A previous prospective study also demonstrated that falls occur prior to cognitive changes among community-dwelling older adults [100]. This prior observation and our current findings support our hypothesis that a recent history of multiple falls may be a biomarker of sub-clinical changes in brain function and increased risk for subsequent decline among community-dwelling older adults without dementia.

We recognize the limitations of our study. The validity of our findings depends on accurate identification of recurrent fallers and non-fallers and previous research has demonstrated that falls recall in older adults is subject to retrospective recall bias [101]. However, we corroborated falls history with immediate family members or close friends. Moreover, fallers had a mean baseline PPA score of 0.60, indicating high physiological falls risk [67]. Our classification scheme separated fallers and non-fallers by 1 fall in the last 12 months (i.e., ≥2 falls versus ≤1 fall) and thus, we may have underestimated the association between falls and disrupted functional connectivity. Future research may wish to classify fallers as those with 3 or more falls in the last 12 months.

Differences in functional connectivity between fallers and non-fallers may be dependent on the specific task imposed. Thus, the generalizability of our findings to other task states will be an important direction of future research. Our functional connectivity analysis was completed with a relatively low number of data points (i.e., 51 per condition) and therefore, may not reflect an ideal signal-to-noise ratio. Potential confounders not accounted for in this study include APOE ε4 genotype; recent evidence report that functional connectivity of large neural networks is influenced by the presence of APOE ε4 allele. We also did not quantify white matter lesions and this should be an important consideration for future functional connectivity research. While we substantially reduced the number of comparison by averaging the ROI-pairs, multiple comparisons were still performed, rendering the results susceptible to Type I error. Notably, results of this exploratory study would no longer be statistically significant if Bonferroni correction (i.e., divide the alpha by the total number of comparisons being made) was applied to our data. Finally, seed-based functional connectivity analysis restricted our results within the scope of the identified regions of interest. Therefore, to extend our results beyond the list of regions and networks presented in this paper, future research should consider data-driven methods of analysis (e.g., Independent Component Analysis).

Our study sample consisted exclusively of independent community-dwelling older adults specifically between the age-range of 70–80 who were without significant physical or cognitive impairment. Thus, our results may not generalize beyond this population of older adults.

In summary, the results of our exploratory study suggest that community-dwelling older adults with a recent history of multiple falls may have disrupted functional connectivity between large neural networks. Importantly, these disruptions *may be* associated with greater decline in both cognitive function and mobility over a 12-month period. Thus, health care professionals working with older adults should consider falls history in their assessment to potentially identify those who are at greater risk for subsequent decline. Lastly, future studies with larger samples will need to be conducted in order to confirm our exploratory findings.

## Supporting Information

Supplementary S1 **Supporting information.** Text S1. Combined Analysis. Table S1. Results from Combined Analysis.
(DOCX)
