# Supplementary material for: Disruptions in Brain Networks of Older Fallers Are Associated with Subsequent Cognitive Decline: A 12-Month Prospective Exploratory Study
Source: PLoS One. 2014 Apr 3;9(4):e93673. doi: 10.1371/journal.pone.0093673 (PMC3977422; doi:10.1371/journal.pone.0093673)
Supplement: Supplementary S1 — Supporting information. Text S1. Combined Analysis. Table S1. Results from Combined Analysis. (DOCX) [file pone.0093673.s001.docx]

Text S1. Combined Analysis

To ensure our results were not affected by the low volume per task condition in the fMRI data, we performed an additional analysis in which all task conditions are combined (to increase the number of time points). Results from this additional analysis are presented in Table 1S below. To further verify whether our original results were affected small number of time points, we have calculated the effect size (Cohen’s d) for the results obtained from the combined analysis and the results from the original analysis.

As indicated by Table 1, the results of the combined analysis concur with our original results. Compared with non-fallers, fallers demonstrated a greater connectivity between the DMN-FPN and less connectivity between the SMN-FPN. We acknowledge the between-group differences in the SMN-FPN were no longer statistically significant in the combined analysis. However, they were trending towards statistical significance (i.e., p < 0.08). Notably, the observed effect sizes for DMN-FPN and SMN-FPN were noticeably greater in our original analysis than those observed in the combined analysis. This suggests what we lost in sampling with our original analysis, we gained in resolution.

Interestingly, the combined analysis produced one significant within-network (i.e., FPN) that was not previously detected. Accordingly, the observed effect size for FPN is greater in the combined analysis than that observed in the original analysis. However, the interpretation of this finding is difficult because of the different conditions (i.e., rest, left finger tapping, and right finger tapping) included in the analysis.

Table S1. Results from Combined Analysis

| **Networks** | **Fallers** | | **Non-Fallers** | | ***p*-value** | **Effect Size of Combined Analysis** | **Effect Size of Original Analysis** |
| --- | --- | --- | --- | --- | --- | --- | --- |
|  | Mean | SD | Mean | SD |  |  |  |
| **DMN-FPN** | -0.14 | 0.10 | -0.19 | 0.11 | 0.03 | 0.48 | 0.63 |
| **Left Hemispheric SMN-FPN** | 0.15 | 0.08 | 0.20 | 0.12 | 0.06 | 0.44 | 0.67 |
| **Right Hemispheric SMN-FPN** | 0.18 | 0.10 | 0.22 | 0.09 | 0.08 | 0.34 | 0.48 |
| **FPN*** | 0.26 | 0.13 | 0.32 | 0.14 | 0.02 | 0.39 | 0.34 |

*denotes new network significantly different between groups after re-analysis
